# The perception of COVID-19, the Light Triad, harmony and ethical sensitivity in late adolescents: The role of meaning-making and stress

**DOI:** 10.1038/s41598-023-35284-4

**Published:** 2023-05-19

**Authors:** Dariusz Krok, Beata Zarzycka, Ewa Telka

**Affiliations:** 1 Institute of Psychology, University of Opole, Plac Staszica 1, 45-052 Opole, Poland; 2 Institute of Psychology, John Paul II Catholic University of Lublin, Lublin, Poland; 3 Maria Sklodowska-Curie National Research Institute of Oncology, Gliwice Branch, Warsaw, Poland

**Keywords:** Psychology, Risk factors

## Abstract

The experiences of inner harmony and ethical sensitivity among late adolescents during the COVID-19 pandemic depend on the interplay of perceptive factors, personal resources and cognitive and stress mechanisms. Using a sample from Poland, the present study examined the relationships between the perceptions of COVID-19 and the Light Triad and the characteristics of inner harmony and ethical sensitivity from the mediational perspective of meaning-making and perceived stress. Three hundred and sixteen late adolescents were recruited in the cross-sectional study. They filled in questionnaires measuring the perception of COVID-19, the Light Triad, meaning-making, stress, inner harmony and ethical sensitivity, from April to September 2020. The perception of COVID-19 was negatively related to ethical sensitivity, whereas the Light Triad was positively related to inner harmony and ethical sensitivity. Perceived stress and meaning-making mediated the relationships between the perceptions of COVID-19, the Light Triad and the characteristic of inner harmony. Perception processes and the Light Triad dimensions directly influence ethical sensitivity, as well as indirectly affect inner harmony through meaning-making processes and perceived stress. This noticeably highlights the vital role played by meaning structures and emotional reactions in the experience of inner peace and calmness.

## Introduction

The COVID-19 pandemic has affected people all over the world and revealed serious psychological problems which underlie everyday life. Unprecedented events have changed our attitudes towards both ourselves and other people. Although coronavirus disease 2019 was initially described as a disease affecting predominately older adults, recent reports have revealed that young people are also at relatively high risk of infection and account for a significant number of confirmed cases^1,2^. While the vast majority of young people did not require hospitalization, they still experienced adverse psychological effects related to the pandemic restrictions. It is thus very important to examine the joint roles of perception of COVID-19 and personal resources in psychosocial outcomes and to understand their underlying mechanisms.

### The explanatory role of protection motivation theory in the perception of the COVID-19 pandemic

The psychological consequences of the COVID-19 pandemic have been examined within a wide range of different theoretical approaches. One of the theory which enables us to more deeply investigate relationships among the perceptions of COVID-19, inner harmony and ethical sensitivity is protection motivation theory (PMT)^3^. It describes the process in which people are motivated to react towards a perceived threat in order to protect their health. Human motivation is analyzed within the following two components: threat appraisal (severity and vulnerability) and coping appraisal (response efficacy/potential costs/self-efficacy). The theory posits that individuals assess the severity of the event, examine the significance of the event from their personal perspective, and respond by using coping appraisal^4^. Threat appraisal comprises the perceived severity of a problematic situation and the perceived probability of its occurrence, while coping appraisal specifies how individuals respond to the situation on a basis self-efficacy that enables them to successfully implement the recommended lines of action. The theory has been effective in examining the effects of fear on individuals’ attitudes and reactions^5^.

Research has confirmed a crucial role of perceptual factors in coping with the psychological consequences of the COVID-19 pandemic. Abdelrahman^6^ found that risk perception was associated with social distancing among the Arab population; individuals who perceived COVID-19 as dangerous were more likely to engage in social distancing practices (e.g., staying at home, avoiding social interactions with relatives and friends or keeping adequate distance). Dryhurst et al.^7^ examined national samples from ten different countries across the world and revealed that risk perception was related to prosocial behavior directed at working for the benefit of others and society. The risk of contracting COVID-19 and its perceived threat were also negatively associated with psychological well-being among healthcare personnel^8^. Taking these studies into account, it is likely that the perception of COVID-19 will be associated with such factors as inner harmony and ethical sensitivity. Inner harmony is a personally-oriented factor which reflects a state of inner peace, self-acceptance, calmness and being in tune with the world, while ethical sensitivity is a socially-oriented factor which describes a person’s attention to ethical values and the ability to recognize that decision-making situations may have ethical content in the realm of social relations^9^ and public health actions during the COVID-10 pandemic^10^. To date, however, there have not been studies examining the direct links among the perceptions of COVID-19, inner harmony and ethical sensitivity.

The main tenets of the PMT found empirical evidence in research demonstrating that SARS survivors’ perceptions and interpretations of the disease were associated with their psychological adjustment^11^. Risk perception of COVID-19 was also negatively related to personal and social dimensions of psychological well-being among healthcare personnel in the early stages of the pandemic^8^. In a large international sample, the way in which coronavirus was perceived predicted judgments and reactions towards other people; higher fear and disgust of coronavirus was related to lower judgments of trustworthiness and lower social desirability^12^. Those studies indicated that the perception of COVID-19 in terms of risk and threat assessment might affect inner harmony and ethical awareness.

### The conservation of resources theory in the context of the Light Triad and psychological reactions to COVID-19

Personal resources also influence the ways in which young people deal with stressful events and maintain well-being. The conservation of resources (COR) theory can be useful to explain how people strive to obtain, retain and nurture personal resources which, in the context of external demands, regulate adaptation processes and determine subsequent outcomes^13^. Developed by Hobfoll, it posits that individuals are motivated to build and nurture personal resources in order to preserve the self and the corresponding social relationships^14^. This is due to the fact that resources are the key components to both forming people’s appraisals of actions as stressful and determining coping responses. Individuals who already lack resources are more likely to experience the loss spirals, but those with plenty of resources are better predisposed to gaining resources. The latter will be more resilient and able to successfully adapt to challenging and stressful events (e.g. the COVID-19 pandemic). Research have confirmed the beneficial role of personal resources in the effective use of coping strategies and in enhancing well-being^15^.

One of the vital personal resources recently developed is the Light Triad which reflects a loving and benevolent orientation towards others^16^. The Light Triad consists of three dimensions: faith in humanity (beliefs in the fundamental goodness of people); humanism (appreciating the dignity and value of every person); and Kantianism (approaching people as ends unto themselves). The very few studies carried out so far showed that the Light Triad was positively related to life satisfaction, personal growth, compassion and empathy and was negatively related to interpersonal aggression, selfishness and anxiety^16^. In addition, higher scores on the Light Triad predicted higher motivation to engage in forming stable and cooperative interpersonal relationships^17^. Malik et al.^18^ also found that individuals who scored high on the Light Triad traits were less likely to demonstrate malevolently creative acts following abusive supervision than those low on the Light Triad. Examining the specific dimensions of the Light Triad and its potential relations to inner harmony and ethical sensitivity can reveal important aspects of the relationship between prosocial behavior and intrapersonal processes. It can show specific functions of faith in humanity, humanism, and Kantianism for harmonious and coherent life experiences.

### Meaning-making and perceived stress as potential mediators

Assessing relationships between threat appraisals and behavioral performance, the PMT further posits that they indirectly occur through mediating factors that include the cognitive and emotional evaluation of the current situation^19^. According to the theory, it can be assumed that late adolescents appraise an event as threatening on the basis of two components: (1) the likelihood of contracting the disease (risk perception of COVID-19) and (2) the assessment of the disease’s perceived severity (perceived threat of COVID-19). These cognitive predictors may have an impact on adolescents’ attitudes to understand and assimilate stressful situations into a coherent framework of personal meaning, beliefs and goals^20^. As a consequence, adolescents will react on a personal level (i.e., inner harmony) and on a social level (i.e., ethical sensitivity) in response to their appraisals of the COVID-19 pandemic.

Meaning-making was found to play a significant mediating role in health-related behavior as it encompasses cognitive activities directed at perceiving and understanding challenging situations in a different way in order to attain consistency among beliefs and goals^21^. As they are connected to motivational structures, meaning-making processes enable individuals to interpret and reorganize stressful events in a positive direction.

Research has shown that meaning-making is associated with more efficient adjustment in the context of stressful events that endanger people’s beliefs and goals^22^. Meaning-making also has also been found to positively contribute to children’s ability to cope with COVID-19 pandemic situations and avoid the traumatic effects of negative cognitive appraisal and emotional reactions^23^. However, disrupted meaning-making has been found to be related to coronavirus anxiety, general anxiety and depression in the general population; additionally, disrupted meaning-making has been shown to mediate the relationship between direct and indirect COVID-19 stressors and mental health consequences^24^. To our knowledge, there have been no studies that have investigated meaning-making as a mediator in the relationship between the perceptions of COVID-19 and the Light Triad with the characteristics of inner harmony and ethical sensitivity in a late adolescent group.

Another mediating factor which may affect personal and social behavior in the context of the COVID-19 pandemic is perceived stress. Within the PMT framework, a person’s intentions which represent a mediating factor can be influenced by his/her general mental state (e.g., emotional reactions)^5^. It is highly likely that stress experienced by late adolescents can exacerbate the way in which they evaluate their thoughts and feelings related to inner harmony and ethical sensitivity. Given that the COVID-19 pandemic has caused a number of stressful conditions for young people (e.g., online learning, restrictions on social gatherings), the mediating effect of stress could be reasonably conspicuous. In fact, a recent study showed that perceived stress was a mediator between the effects of the COVID-19 pandemic and well-being among both parents and children^25^. Stress also mediated associations between perceptions of social support and physical and mental well-being in American undergraduates^26^ and between emotional intelligence and psychological well-being in Spanish postgraduates^27^. In addition, stress was a mediator in the relationship between personality traits (neuroticism, agreeableness and conscientiousness) and negative attitudes towards oneself among Dutch and Belgian adolescents^28^. However, there has not thus far been research that would directly examine a mediating role of stress in the context of the COVID-19 pandemic, the Light Triad and personally- and socially-oriented factors in adolescents, thus leaving a gap in the existing knowledge on potential mechanisms underlying the experiences of inner harmony and ethical sensitivity.

### The present study

The purpose of the present study is to examine the relationship between the perceptions of COVID-19 and the dimensions of the Light Triad with the characteristics of inner harmony and ethical sensitivity from the mediational perspective of meaning-making and perceived stress based on a sample from Poland. Based on the PMT and existing research, four hypotheses have been formulated: (1) a higher perception of COVID-19 would be associated with higher inner harmony and ethical sensitivity, (2) the Light Triad would be positively associated with inner harmony and ethical sensitivity; (3) meaning-making and perceived stress would mediate the association of the perception of COVID-19 with inner harmony and ethical sensitivity; and (4) meaning-making and perceived stress would mediate the association of the Light Triad with inner harmony and ethical sensitivity. However, the mediating effects of meaning-making and stress would be different due to their contrasting character.

## Material and methods

### Power analysis

As we did not conduct a priori power analysis for SEM, we decided to compute post hoc power analysis for RMSEA through R programming language (package “semPower”, function “semPower.postHoc”)^29^. Taking in the account the size of the sample used in our study (N = 316), post hoc power (1-β) for RMSEA was 0.99 (effect = 0.08, effect measure = RMSEA, Beta = 0.01, Alpha = 0.05, N = 316, df = 19). The sample was thus sufficient for our study.

### Participants and procedure

Three hundred and sixteen late adolescents (173 women and 143 men) participated in this study, from April to September 2020. The participants’ ages ranged from 17 to 24 with the mean age of 21.58 (SD = 2.03). All of them were Caucasians who constituted a representative sample of Polish late adolescents in terms of gender, age and socio-cultural background. To ensure the representativeness of the group, we used official statistics published by the Central Statistical Office in our country^30^. The study was anonymous and participants were recruited via personal contact, colleges and cultural organizations. They were invited to fill in a set of questionnaires which were then either collected by research assistants or sent back by post. The participants were able to quit the study at any time, and after the study, they were fully debriefed. Informed consent was obtained from either participants or their parents (for those who were underage) in accordance with ethical standards approved by the University Ethics Committee.

### Measures

#### Risk of contracting COVID-19

Perceptions of risk and knowledge of contracting COVID-19 were evaluated with The Risk of Contracting COVID-19 Scale which was developed in Polish^8^. It consists of 10 items rated on a 5-point Likert scale from 1 (strongly disagree) to 5 (strongly agree). Sample items are: ‘Getting infected with coronavirus is dangerous to my health’; ‘I am worried that I may not be able to protect myself from getting COVID-19’. A high score represents a higher perceived likelihood of contracting coronavirus. The Cronbach’s α reliability for the current study was 0.86.

#### Perceived threat of COVID-19

Perceptions of threat severity of coronavirus were assessed with The Perceived Threat of COVID-19 Scale which was developed in Polish^8^. It comprises six items rated on a 5-point Likert scale from 1 (strongly disagree) to 5 (strongly agree). The scale reflects the negative personal, societal and economic consequences that people associate with the coronavirus pandemic. Sample items are: ‘The coronavirus pandemic causes serious health problems’; ‘The coronavirus pandemic negatively affects social relations’. The Cronbach’s α reliability for the current study was 0.82.

#### The Light Triad

The Light Triad Scale developed by Kaufman et al. is a 12-item questionnaire that measures three dimensions: Faith in humanity (e.g., ‘I tend to see the best in people’; ‘I think people are mostly good’), Humanism (e.g., ‘I tend to treat others as valuable’; ‘I enjoy listening to people from all walks of life’) and Kantianism (e.g., ‘I don’t feel comfortable overtly manipulating people to do something I want’; ‘I would like to be authentic even if it may damage my reputation’)^16^. The scale assesses a caring and beneficent disposition towards other people. Items are rated on a 5-point Likert scale from 1 (strongly disagree) to 5 (strongly agree). The scale was adapted into Polish by Gerymski and Krok^31^. The Cronbach’s α reliability coefficients for the current study were 0.78 (Faith in Humanity), 0.69 (Humanism) and 0.63 (Kantianism).

#### Inner harmony

The Inner Harmony Scale is part of The Self-description Questionnaire, which was developed in Polish^32^, measures a mental state of self-acceptance, emotional stability, balance and acceptance of one's life in general. It includes seven items rated on a 5-point Likert scale from 1 (definitely not) to 5 (definitely yes). Sample items are: ‘I feel deep inner peace’; ‘Thinking about my life I experience peace and joy’. The Cronbach’s alpha coefficient for the present study was 0.83.

#### Ethical sensitivity

The Ethical Sensitivity Scale, which was developed in Polish, evaluates a person’s consideration for ethical and moral values and the ability to respond to a situation in a moral way^32^. It comprises seven items rated on a 5-point Likert scale from 1 (definitely not) to 5 (definitely yes). Sample items are: ‘I react when someone is being harmed’; ‘I care about other people’. The Cronbach’s alpha coefficient for the present study was 0.82.

#### Meaning-making

The Meaning-Making Questionnaire, which was developed in Polish, evaluates one’s cognitive capacity to understand and assimilate challenging or ambiguous situations into a coherent framework of personal meaning, beliefs and goals^8^. It includes eight items rated on a 5-point Likert scale ranging from 1 (never) to 5 (very often). A higher score reflects a stronger process of meaning-making. Sample items are: ‘I search for something that is really important in my life’; ‘I focus on those aspects of my life which enable me to find meaning in life’. The Cronbach’s alpha coefficient for the present study was 0.88.

#### Stress

The Perceived Stress Scale is a 10-item tool that assesses the level of stress perceived by individuals. It was developed by Cohen et al.^33^ and adapted into Polish by Juczynski and Oginska-Bulik^34^. It consists of 10 items which are rated on a 5-point Likert scale ranging from 0 (never) to 4 (very often). Higher scores indicate a higher level of perceived stress. Sample items are: ‘Have you been upset because of something that happened unexpectedly?’; ‘Have you felt nervous and “stressed”’. The Cronbach’s alpha coefficient for the present study was 0.78.

### Statistical methods

A cross-sectional design was used in the current study. All analyses were carried out in SPSS Statistics 21 and AMOS 21. First, descriptive statistics and correlational analyses for all the variables were performed to examine relationships among the perception of COVID-19, the Light Triad dimensions, inner harmony, ethical sensitivity, meaning-making and stress. Second, structural equation modeling (SEM) with bootstrapping (95% bias-corrected confidence intervals, 5,000 bootstrap re-samples)^35^ was used to examine the potential mediational effects of meaning-making and stress in the relationship between the perceptions of COVID-19 and the Light Triad with the characteristics of inner harmony and ethical sensitivity. Both direct and indirect effects among the variables were calculated.

### Ethics approval

All procedures performed in our study involving human subjects were in accordance with the ethical standards of the institutional and/or national research committee and with the 1964 Helsinki declaration and its later amendments or comparable ethical standards. The University Research Ethics Committee at The University of Opole approved the study ‒ the reference number for the approval: UREC ‒ UO/04/2020.

## Results

Most of the correlational coefficients turned out to be statistically significant (Table 1). Their strength was small or moderate in most cases. Age was negatively associated with perceived threat of COVID-19, humanism, and Kantianism. Risk of contracting and perceived threat of COVID-19 were positively related to humanism, Kantianism, ethical sensitivity and meaning-making. Perceived threat of COVID-19 was also positively related to faith in humanity and stress. All the domains of the Light Triad, namely faith in humanity, humanism and Kantianism, were positively correlated to inner harmony, ethical sensitivity and meaning-making. In contrast, faith in humanity, meaning-making and inner harmony had negative associations with stress. Interestingly, there was no significant correlation between the mediating variables of meaning-making and stress, which supports their rather independent character.

**Table 1:** Means, standard deviations and correlations among age, risk of contracting COVID-19, perceived threat of COVID-19, the Light Triad dimensions, inner harmony, ethical sensitivity, meaning-making and stress.

Variables	1	2	3	4	5	6	7	8	9	10
1. Age	‒									
2. Risk of contracting COVID-19	− 0.07	‒								
3. Perceived threat of COVID-19	− 0.14*	0.65***	‒							
4. Faith in Humanity	− 0.08	0.09	0.17**	‒						
5. Humanism	− 0.18**	0.17**	0.26***	0.51***	‒					
6. Kantianism	− 0.17**	0.23***	0.33***	0.47***	0.59***	‒				
7. Inner harmony	− 0.04	0.05	0.05	0.44***	0.32***	0.29***	‒			
8. Ethical sensitivity	− 0.07	0.27**	0.34***	0.42***	0.46***	0.54***	0.41***	‒		
9. Meaning-making	− 0.07	0.16**	0.16**	0.28***	0.44***	0.42***	0.48***	0.39***	‒	
10. Perceived stress	− 0.03	0.10	0.13*	− 0.18**	− 0.06	− 0.05	− 0.45***	0.02	− 0.27***	‒
*M*	21.58	3.78	4.15	3.31	3.76	3.86	3.31	3.84	3.61	2.05
*SD*	2.06	0.80	0.73	0.81	0.70	0.74	0.79	0.69	0.87	0.64

* *p* < 0.05; ** *p* < 0.01; *** *p* < 0.001.

### Mediational effects of meaning-making and perceived stress

Structural equation modelling (SEM) with bootstrapping was used to examine the mediational effects of meaning-making and stress in the relationship between the perception of COVID-19 and the Light Triad with inner harmony and ethical sensitivity. The measurement model including two latent factors (the perception of COVID-19 and the Light Triad) and five observed variables was calculated. The confirmatory factor analysis revealed a very satisfactory fit to the data: *χ*2 (N = 316) = 74.25, *p* < 0.001; GFI = 0.92; CFI = 0.90; NFI = 0.91; RMSEA = 0.05; SRMR = 0.04. All the factor loadings for the indicators on the two latent factors were significant (*p* < 0.001; values ranged from 0.70 to 0.85).

Next, we tested a model which included directional paths between the variables. The initial model with two mediators (meaning-making and perceived stress) did not show an adequate fit to the data: *χ*2 (19, N = 316) = 93.98, *p* < 0.001; RMSEA = 0.12; GFI = 0.89; CFI = 0.71; SRMR = 0.11; Hoelter's index = 105. Furthermore, the model included some statistically non-significant paths.

**Figure 1:**
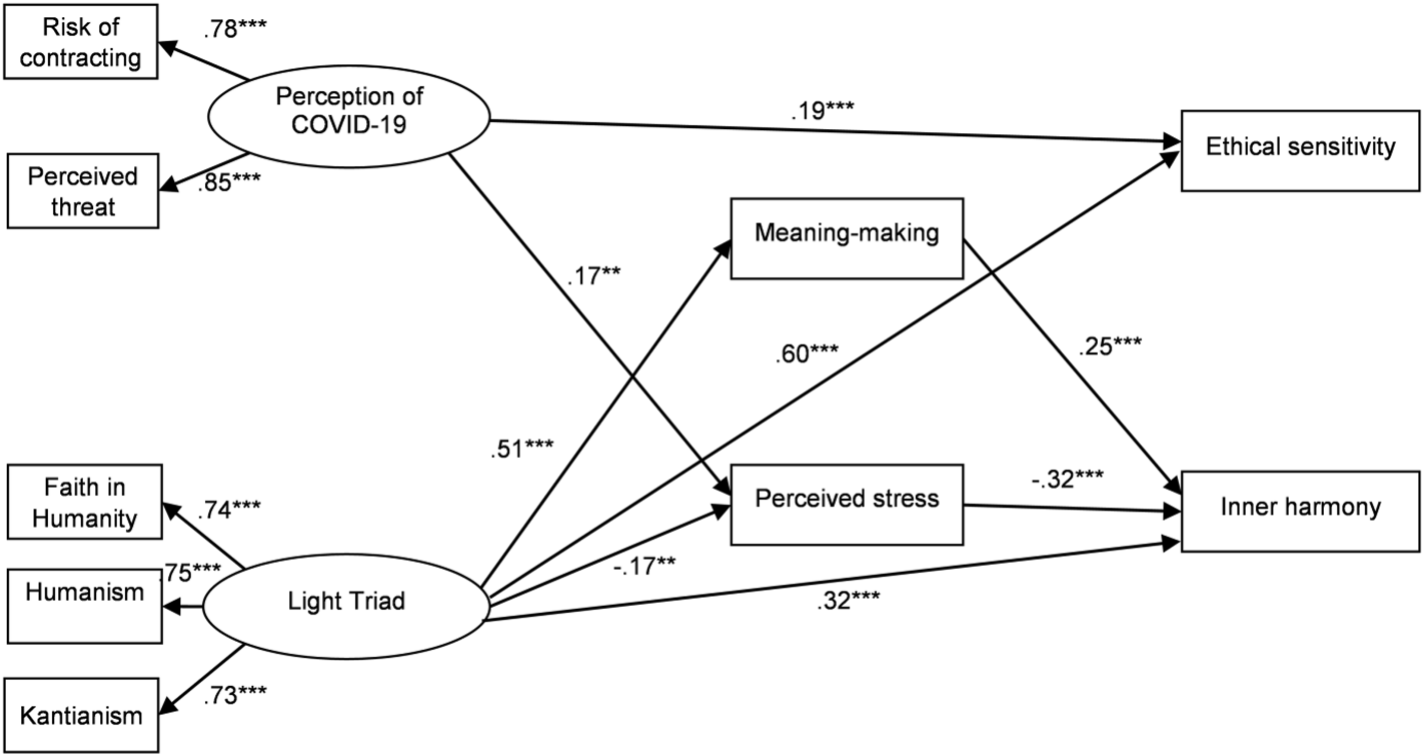
The final mediational model of the relationships between the perception of COVID-19, the Light Triad, meaning-making, perceived stress, ethical sensitivity and inner harmony (standardised coefficients) ** *p *< .01; *** *p *< .001.

The initial model was then re-tested in accordance with procedures based on modification indices and estimates. This resulted in a final model which demonstrated a satisfactory fit to the data: *χ*2 (21, N = 316) = 71.05, *p *< 0.005; RMSEA = 0.07; GFI = 0.95; CFI = 0.85; SRMR = 0.06; Hoelter's index = 205. All the direct and indirect paths included in the final model were significant (Fig. 1). The comparison of the final model with the initial model confirmed a more satisfactory fit of the former: Δ*χ*2 (3, N = 316) = 22.93, *p* < 0.001.

Next, the final model was tested for the mediational effects, using bootstrapping as recommended by Hayes^35^. The results of indirect effects and confidence intervals are presented in Table 2.

**Table 2:** Bootstrapping standardised estimates and 95% confidence intervals for the final model.

Model pathways	Estimate	95% CI	
		Lower	Upper
Perception of COVID-19 → Perceived stress → Inner harmony	− 0.06^a^	− 0.10	− 0.01
Light triad → Meaning-making/Perceived stress → Inner harmony	0.18^a^	0.11	0.27

^a^Empirical 95% confidence interval does not overlap with zero.

The final model included three direct effects: from the perception of COVID-19 on ethical sensitivity and from the Light Triad on ethical sensitivity and on inner harmony. There were also significant indirect effects from the perception of COVID-19 on inner harmony through perceived stress and from the Light Triad on inner harmony through meaning-making and perceived stress. Their effect sizes were rather weak (− 0.06, 0.18). In contrast, there were no indirect effects from either the perception of COVID-19 or the Light Triad on ethical sensitivity. Meaning-making and perceived stress were thus mediators only in regard to inner harmony.

Additionally, we tested SEM models with the Light Triad dimensions individually: faith in humanity, humanism, and Kantianism as separate variables (the entire analysis is included in Supplementary material). The final models had a satisfactory fit to the data: (1) the model with faith in humanity: *χ*^2^ (8, N = 316) = 19.17, *p *< 0.001; RMSEA = 0.07; GFI = 0.98; CFI = 0.94; SRMR = 0.07; Hoelter's index = 248; (2) the model with humanism: *χ*^2^ (9, N = 316) = 26.09, *p *< 0.001; RMSEA = 0.08; GFI = 0.97; CFI = 0.92; SRMR = 0.08; Hoelter's index = 262; and (3) the model with Kantianism: *χ*^2^ (9, N = 316) = 36.45, *p *< 0.001; RMSEA = 0.09; GFI = 0.96; CFI = 0.89; SRMR = 0.08; Hoelter's index = 201. All the three models including faith in humanity, humanism, and Kantianism, respectively had three direct effects: from the perception of COVID-19 on ethical sensitivity and from faith in humanity, humanism, and Kantianism on ethical sensitivity and on inner harmony. There was thus no difference in direct effects between the model with all the Light Triad dimensions grouped as one latent variable and the models with the Light Triad dimensions tested individually. However, the models slightly differed in terms of indirect effects. In the models with faith in humanity and humanism, meaning-making and perceived stress were mediators for ethical sensitivity and inner harmony, whereas in the model with Kantianism, meaning-making and perceived stress were mediators only for inner harmony. Therefore, the models for faith in humanity and humanism, but not the model with Kantianism as independent variables differed from the general model. This comparison showed that meaning-making and perceived stress are mediators not only for the relationship of faith in humanity and humanism with inner harmony, but also with ethical sensitivity. In contrast, the mediating effects for Kantianism occur only in the case of inner harmony.

Finally, we set out to test whether stress and meaning-making could moderate the relationship between the perception of COVID-19, the Light Triad (independent variables), and inner harmony and ethical sensitivity (dependent variables). However, the overall model had an unsatisfactory fit with *χ*2 = 102.31, *p* < 0.001; RMSEA = 0.19; GFI = 0.73; CFI = 0.701; SRMR = 0.12. Finally, a multi-group analysis was conducted to examine potential differences in the mediational models between women and men. Its results demonstrated that the path coefficients for meaning-making and perceived stress as mediators were non-statistical across gender (χ2 = 1.12, *p* > 0.05).

## Discussion

The aim of the present study was to examine the relationship between the perceptions of COVID-19 and the Light Triad with the characteristics of inner harmony and ethical sensitivity based on a sample from Poland. In addition, the study aimed to investigate whether meaning-making and perceived stress mediated those relationships. To our knowledge, this is the first study to evaluate an integrated mediational model including such variables in the context of the COVID-19 pandemic. The findings supported most of our hypotheses, suggesting their relevance in understanding how young people react in the domains of harmony and ethical values in the time of coronavirus.

### Associations between the perception of COVID-19 and ethics and harmony

With regard to our first hypothesis, we found that a higher level of the perception of COVID-19 was associated with higher ethical sensitivity but not with inner harmony. This partially supports this hypothesis as it also assumed the latter association. Young people who more intensively perceive the COVID-19 pandemic in terms of serious coronavirus contraction risk and apparent threat tend to pay more attention to ethical values and decisions shown in social relations. This is in line with prior research which demonstrated that the way in which COVID-19 was perceived was related to moral judgments and reactions towards others^12^. Yet our findings extend the existing knowledge by revealing that young people, despite the widespread opinions of their disregard for safety rules and imposed social restrictions, nevertheless show ethical responsibility in their relations with others. Awareness of the risk of coronavirus infection and the economic and cultural threat to society makes young people sensitive to observing ethical principles and norms in their daily lives^25^. This situation may at least partly result from the fact that young people have older parents or relatives who are more at risk of coronavirus infection. In addition, age was negatively associated with perceived threat of COVID-19, humanism, and Kantianism. It suggests that younger adolescents pay less attention to threats posed by COVID-19, show less concern for the dignity of every person, and more objectify people.

To our surprise, the perception of COVID-19 was not associated with inner harmony. This stands in contrast with previous research which indicated that Hong Kong SARS survivors’ perceptions of the disease were related to their psychological adjustment^11^. We can point to two important elements to try to explain this result. First, the SARS-CoV-2 pandemic has had a much broader scope and stronger social, cultural and economic consequences than the SARS pandemic, which means that people’s psychological reactions may be different^2^. Second, there are undeniable cultural differences between the approach of the European and Chinese populations to experiencing the states of inner harmony in terms of calmness, self-acceptance and composure. In comparison with European cultures, the cultures of East Asia more strongly emphasise the need for inner peace and harmony of mind and body and appreciate the state of calm and composure^36^. As a consequence, the perception of COVID-19 may be less linked to the experiences of internal harmony in young people in Europe than in their Chinese colleagues.

### The light triad and ethics and harmony

The second hypothesis that assumed positive associations between the Light Triad, inner harmony and ethical sensitivity was supported. All three of the domains, namely faith in humanity, humanism and Kantianism, were positively related to inner harmony and ethical sensitivity. These results suggest that late adolescents who treat people as subjects, value their dignity and worth and believe in human goodness are characterized by a state of inner peace and self-acceptance as well as a conscientious adherence to ethical standards and principles. This pattern is fully understood within the framework of the conservation of resources theory^13^; young people’s personal resources based on an idiosyncratic and humanistic approach to others facilitate their internal harmony and regulate adaptation to the social environment. This may be due to the natural developmental characteristics of late adolescents who express themselves in the pursuit of noble ideals and valuable goals and fulfilment of idealistic intentions, which can be especially important in the context of reappraisal interventions during stressful events^2,37,38^.

### The mediating role of meaning-making and stress

The main findings of our study reveal the mediating effects of meaning-making and perceived stress. The third hypothesis which proposed that meaning-making and perceived stress would mediate the association of the perception of COVID-19 with inner harmony and ethical sensitivity was partially supported. Perceived stress, but not meaning-making, was a mediator between the perception of COVID-19 and inner harmony. Specifically, the perception of the pandemic was related to a higher level of stress which, in turn, was associated with lower inner harmony. This is consistent with previous research among American undergraduates in whom stress mediated associations between the perception of social support and well-being^26^. Stress was also found to mediate the relationship of the Light Triad with inner harmony; the relationship was mediated by meaning-making, which was assumed by the fourth hypothesis. Additionally, in the models with faith in humanity and humanism, respectively, meaning-making and perceived stress were mediators for ethical sensitivity and inner harmony, whereas in the model with Kantianism, meaning-making and perceived stress were mediators only for inner harmony. These findings concur with prior empirical studies which have demonstrated the predictive validity of meaning-making as a mediator for understanding people’s reactions towards stressful events caused by natural disasters^22^ or the COVID-19 pandemic^23^. They also support the important role of understanding challenging and stressful situations in the light of meaning and purpose.

Our findings also shed new light on young people’s psychological reactions to the COVID-19 pandemic and their underlying mechanisms related to meaning-making and stress. Due to its ability to perceive and understand difficult situations in a different way, meaning-making turned out to be a highly beneficial cognitive pathway from the Light Triad to enhancing and developing in young people a state of inner peace and calmness; in our study, meaning-making thus had a positive character. Therefore, it expands on Milman et al.’s research on disruptive meaning-making efforts by demonstrating that productive meaning-making as a mediator is associated with higher inner harmony in young people affected by the COVID-19 pandemic^24^. During this difficult period, the meaning-making process enables adolescents to reduce insecurity and fear and restore a sense of comprehensibility and purposefulness^39,40^, which leads to inner peace and calmness.

From the PMT theory perspective^19^, meaning-making may operate as a mediating factor involving the cognitive re-evaluation of the current situation due to its relation to the domains of purpose and goals. Young people who can effectively construct meaning by interpreting the COVID-19 unprecedented stressful events, revising important goals and seeking a deeper sense tend to achieve a satisfactory level of inner harmony. This interpretation is supported by previous research conducted by Provenzi et al. in which meaning-making was conducive to children’s coping abilities during the COVID-19 pandemic due to its relations with the domains of goals and hope^23^.

The second mediator investigated in our study, that is, perceived stress, had the opposite effect; the perception of COVID-19 was related to higher stress and the Light Triad was related to lower stress which, in turn, was associated with lower inner harmony. These results are in line with studies which pointed to stress as a mediator between the effects of the COVID-19 pandemic and well-being^24^ and between personal resources and well-being^26^. Yet these results also expand our knowledge by revealing the underlying mediational function of stress in the association among late adolescents of the perception of COVID-19 and the Light Triad with experiences of inner harmony. Namely, decreasing the negative perception of COVID-19 and increasing a loving and benevolent orientation towards others may indirectly strengthen young people’s states of inner peace and calmness by reducing the experience of stress. Young people who have an objective knowledge of COVID-19 risk and a prosocial attitude towards others may more successfully manage stressful epidemic situations, which would in turn lead to higher inner harmony. This interpretation broadens the COR theory by indicating that young people not only conserve or protect their valued resources to reduce the likelihood of stress^13^, but also do so in conjunction with a cognitive assessment of potential risk factors for COVID-19^40,41^.

### Limitations

Our findings have several limitations. First, due to the cross-sectional nature of the study, the causality of any relationships between variables cannot be determined. Further research is required to examine the causal mechanisms by which young people’s perception of COVID-19 and positive attitudes towards others may reduce their levels of stress and subsequently increase inner harmony. Second, although we examined meaning-making and stress as mediators, some other psychological constructs, such as personality traits^3^ or coping strategies^8^, may also influence the relationship of the perception of COVID-19 and the Light Triad with the characteristics of inner harmony and ethical sensitivity; these relationships can be also affected by the fact that two dimensions of the Light Triad (i.e. humanism and Kantianism) did not reach the conventional reliability levels (α < 0.70) (their respective values were 0.69 and 0.63). Third, our data collection was done through a 5-month period which was a long time for the pandemic. Therefore, the dynamically changing health, epidemiological, and legal situations could undoubtedly have influenced the results of our study.

### Implications and conclusion

Despite these limitations, the current study compellingly showed the significance of investigating the relationships between the perception of COVID-19 and the Light Triad with personal- and social-oriented factors in late adolescents within a mediation model incorporating meaning-making and stress. Our results make it possible to formulate some essential implications. In the context of the traumatic events associated with the COVID-19 pandemic, both perceptual and prosocial orientation factors influence late adolescents’ abilities to maintain inner composure and ethical judgment. Furthermore, meaning-making processes and emotional reactions play a significant role in the way in which young people achieve balance of mind and self-control during the times of COVID-19^2,20^. As the mediation effects revealed, it is primarily the interaction of cognitive and emotional processes that determines the extent of attention paid to ethical values and decision-making in social relationships. Therefore, young people are able to construct a satisfying and coherent vision of their lives based on a constructive assessment of both the pandemic reality and intrapsychic processes. Our results obtained on late adolescents also provide an inspiration for future research to test whether the results could have been different in other age groups.

## Supplementary Information

Supplementary Information.

## Data Availability

The database and study materials for this article can be found at the OSF HOME repository, https://osf.io/2wvf8/.
