# Supplementary material for: The perception of COVID-19, the Light Triad, harmony and ethical sensitivity in late adolescents: The role of meaning-making and stress
Source: Sci Rep. 2023 May 19;13:8122. doi: 10.1038/s41598-023-35284-4 (PMC10196279; doi:10.1038/s41598-023-35284-4)
Supplement: Supplementary file 1 — Supplementary Information. [file 41598_2023_35284_MOESM1_ESM.docx]

**The perception of COVID-19, the Light Triad, harmony and ethical sensitivity in late adolescents: The role of meaning-making and stress**

**SUPPLEMENTARY MATERIAL**

We tested SEM models with the Light Triad dimensions individually: faith in humanity, humanism, and Kantianism as separate variables. Therefore, three different models were tested with the following variables:

Independent variables: the perception of COVID-19 and faith in humanity, humanism, and Kantianism (the last three variables as separate ones).

Mediators: meaning-making and stress

Dependent variables: inner harmony and ethical sensitivity

**1) Faith in humanity as independent variable**

Structural equation modelling (SEM) with bootstrapping was used to examine the mediational effects of meaning-making and stress in the relationship between the perception of COVID-19 and faith in humanity with inner harmony and ethical sensitivity. The measurement model including one latent factors (the perception of COVID-19) and five observed variables was calculated. The confirmatory factor analysis showed a very satisfactory fit to the data: *χ*2 (N = 316) = 89.25, *p* < .001; GFI = .93; CFI = .91; NFI = .92; RMSEA = .04; SRMR = .03. All the factor loadings for the indicators on the two latent factors were significant (*p* < .001; values ranged from .74 to .89).

Next, we tested a model which included directional paths between the variables. The initial model with two mediators (meaning-making and perceived stress) did not show an adequate fit to the data: *χ*2 (7, N = 316) = 52.78, *p* < .001; RMSEA = .15; GFI = .88; CFI = .78; SRMR = .08; Hoelter's index = 111. Furthermore, the model included some statistically non-significant paths.

We decided to retest the initial model in accordance with procedures based on modification indices and estimates. This resulted in a final model which demonstrated a satisfactory fit to the data: *χ*2 (8, N = 316) = 19.17, p < .001; RMSEA = .07; GFI = .98; CFI = .94; SRMR = .07; Hoelter's index = 248. All the direct and indirect paths included in the final model were significant (Figure 1). The comparison of the final model with the initial model confirmed a more satisfactory fit of the former: Δ*χ*2 (1, N = 316) = 33.61, *p* < .001.

Figure 1. The final mediational model of the relationships between the perception of COVID-19, faith in humanity, meaning-making, perceived stress, ethical sensitivity, and inner harmony (standardised coefficients)

*** p < .001

Next, the final model was tested for the mediational effects, using bootstrapping as recommended by Hayes^35^. The results of indirect effects and confidence intervals are presented in Table 1.

**Table 1**

Bootstrapping standardised estimates and 95% confidence intervals for the final model.

| Model pathways | Estimate | 95% CI | |
| --- | --- | --- | --- |
|  |  | Lower | Upper |
| Perception of COVID-19 → Perceived stress → Inner harmony | -.08^a^ | -.12 | -.04 |
| Faith in humanity → Meaning-making/Perceived stress → Ethical sensitivity | .07^a^ | .03 | .10 |
| Faith in humanity → Meaning-making/Perceived stress → Inner harmony | .14^a^ | .07 | .22 |

^a^ Empirical 95% confidence interval does not overlap with zero.

The final model included three direct effects: from the perception of COVID-19 on ethical sensitivity and from faith in humanity on ethical sensitivity and on inner harmony. There were also significant indirect effects from the perception of COVID-19 on inner harmony through perceived stress and from faith in humanity on ethical sensitivity and inner harmony through meaning-making and perceived stress. Their effect sizes ranged from -.08 to .14. Perceived stress was a mediator between perception of COVID-19 and inner harmony, whereas meaning-making and perceived stress were mediators between faith in humanity and ethical sensitivity and inner harmony.

Finally, a multi-group analysis was conducted to examine potential differences in the mediational models between women and men. Its results demonstrated that the path coefficients for meaning-making and perceived stress as mediators were non-statistical across gender (χ2 = 1.05, *p* > .05).

**2) Humanism as independent variable**

Next, structural equation modelling (SEM) with bootstrapping was used to examine the mediational effects of meaning-making and stress in the relationship between the perception of COVID-19 and humanism with inner harmony and ethical sensitivity. The measurement model including one latent factors (the perception of COVID-19) and five observed variables was calculated and its confirmatory factor analysis showed a very satisfactory fit to the data: *χ*2 (N = 316) = 83.79, *p* < .001; GFI = .93; CFI = .92; NFI = .93; RMSEA = .05; SRMR = .05. All the factor loadings for the indicators on the two latent factors were significant (*p* < .001; values ranged from .75 to .86).

Next, we tested a model which included directional paths between the variables. The initial model with two mediators (meaning-making and perceived stress) did not show an adequate fit to the data: *χ*2 (7, N = 316) = 76.21, *p* < .001; RMSEA = .18; GFI = .90; CFI = .69; SRMR = .09; Hoelter's index = 77. Furthermore, the model included some statistically non-significant paths.

The initial model was then retested based on modification indices and estimates. As a result, the final model showed a satisfactory fit to the data: *χ*2 (9, N = 316) = 26.09, p < .001; RMSEA = .08; GFI = .97; CFI = .92; SRMR = .08; Hoelter's index = 262. All the direct and indirect paths included in the final model were significant (Figure 2). The comparison of the final model with the initial model confirmed a more satisfactory fit of the former: Δ*χ*2 (2, N = 316) = 50.12, *p* < .001.

Figure 2. The final mediational model of the relationships between the perception of COVID-19, humanism, meaning-making, perceived stress, ethical sensitivity, and inner harmony (standardised coefficients)

** p < .01; *** p < .001

The final model was calculated to verify mediational effects^35^ and its results are shown in Table 2.

**Table 2**

Bootstrapping standardised estimates and 95% confidence intervals for the final model.

| Model pathways | Estimate | 95% CI | |
| --- | --- | --- | --- |
|  |  | Lower | Upper |
| Perception of COVID-19 → Perceived stress → Inner harmony | -.08^a^ | -.14 | -.03 |
| Humanism → Meaning-making/Perceived stress → Ethical sensitivity | .08^a^ | .04 | .12 |
| Humanism → Meaning-making/Perceived stress → Inner harmony | .11^a^ | .06 | .17 |

^a^ Empirical 95% confidence interval does not overlap with zero.

In a similar way as with the previous variable (i.e. faith in humanity), the final model with humanism included three direct effects: from the perception of COVID-19 on ethical sensitivity and from humanism on ethical sensitivity and on inner harmony. There were also significant indirect effects from the perception of COVID-19 on inner harmony through perceived stress and from humanism on ethical sensitivity and inner harmony through meaning-making and perceived stress. Their effect sizes ranged from -.08 to .11. Meaning-making and perceived stress were mediators for ethical sensitivity and inner harmony.

Finally, a multi-group analysis which was to examine potential differences in the mediational models between women and men showed that the path coefficients for meaning-making and perceived stress as mediators were non-statistical across gender (χ2 = 1.19, *p* > .05).

**3) Kantianism as independent variable**

For the last dimension of the Light Triad, structural equation modelling (SEM) with bootstrapping was used to examine the mediational effects of meaning-making and stress in the relationship between the perception of COVID-19 and Kantianism with inner harmony and ethical sensitivity. The measurement model including one latent factors (the perception of COVID-19) and five observed variables was calculated and its confirmatory factor analysis showed a very satisfactory fit to the data: *χ*2 (N = 316) = 79.18, *p* < .001; GFI = .91; CFI = .90; NFI = .91; RMSEA = .07; SRMR = .06. All the factor loadings for the indicators on the two latent factors were significant (*p* < .001; values ranged from .73 to .84).

Next, we tested a model which included directional paths between the variables. The initial model with two mediators (meaning-making and perceived stress) did not show an adequate fit to the data: *χ*2 (7, N = 316) = 87.74, *p* < .001; RMSEA = .19; GFI = .90; CFI = .65; SRMR = .10; Hoelter's index = 67. The model also included statistically non-significant paths.

We recalculated the initial model in line with modification indices and estimates. As a result, the final model showed a satisfactory fit to the data: *χ*2 (9, N = 316) = 36.45, *p* < .001; RMSEA = .09; GFI = .96; CFI = .89; SRMR = .08; Hoelter's index = 201. All the direct and indirect paths included in the final model were significant (Figure 3). The comparison of the final model with the initial model confirmed a more satisfactory fit of the former: Δ*χ*2 (2, N = 316) = 51.29, *p* < .001.

Figure 3. The final mediational model of the relationships between the perception of COVID-19, Kantianism, meaning-making, perceived stress, ethical sensitivity, and inner harmony (standardised coefficients)

* p < .05; *** p < .001

The final model was retested to check mediational effects^35^ which are presented in Table 3.

**Table 3**

Bootstrapping standardised estimates and 95% confidence intervals for the final model.

| Model pathways | Estimate | 95% CI | |
| --- | --- | --- | --- |
|  |  | Lower | Upper |
| Perception of COVID-19 → Perceived stress → Inner harmony | -.08^a^ | -.14 | -.04 |
| Kantianism → Meaning-making/Perceived stress → Inner harmony | .11^a^ | .06 | .16 |

^a^ Empirical 95% confidence interval does not overlap with zero.

The final model with Kantianism included three direct effects: from the perception of COVID-19 on ethical sensitivity and from humanism on ethical sensitivity and on inner harmony. There were also two significant indirect effects: (1) from the perception of COVID-19 on inner harmony through perceived stress and (2) from Kantianism on inner harmony through meaning-making and perceived stress. Their effect sizes ranged were -.08 and .11, respectively. Perceived stress was a mediator for inner harmony, whereas meaning-making and perceived stress were also mediators for inner harmony.

Finally, a multi-group analysis was conducted to check differences between women and men. Yet, it demonstrated that the path coefficients for meaning-making and perceived stress as mediators were non-statistical across gender (χ2 = 1.23, *p* > .05).
